# Bis(*N*-isobutyl-*N*-propyl­dithio­carbamato-κ^2^ *S*,*S*′)zinc(II)

**DOI:** 10.1107/S1600536810002825

**Published:** 2010-01-30

**Authors:** Norma Awang, Ibrahim Baba, Bohari M. Yamin, Seik Weng Ng

**Affiliations:** a School of Chemical Sciences, Universiti Kebangbaan Malaysia, 43600 Bangi, Malaysia; b Department of Chemistry, University of Malaya, 50603 Kuala Lumpur, Malaysia

## Abstract

In the title compound, [Zn(C_8_H_16_NS_2_)_2_], the Zn^II^ atom is chelated by two *S*,*S*′-bidentate dithio­carbamate ions in a very distorted tetra­hedral geometry. The alkyl chains of the ligands are equally disordered over two sets of sites.

## Related literature

For other monomeric zinc bis­(dithio­carbamates), see: Chan *et al.* (2004 ▶); Cox & Tiekink (1999 ▶); Decken *et al.* (2004 ▶); Reck & Becker (2003 ▶); Zhong *et al.* (2003 ▶).
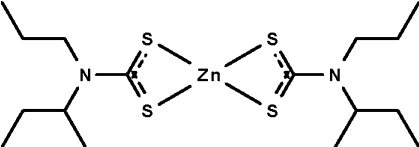


## Experimental

### 

#### Crystal data

[Zn(C_8_H_16_NS_2_)_2_]
*M* *_r_* = 446.05
Monoclinic, 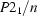

*a* = 14.2151 (7) Å
*b* = 11.8527 (6) Å
*c* = 15.1428 (7) Å
β = 115.691 (1)°
*V* = 2299.15 (19) Å^3^
*Z* = 4
Mo *K*α radiation
μ = 1.43 mm^−1^
*T* = 293 K
0.30 × 0.30 × 0.25 mm

#### Data collection

Bruker SMART APEX diffractometer
Absorption correction: multi-scan (*SADABS*; Sheldrick, 1996 ▶) *T*_min_ = 0.673, *T*_max_ = 0.716
15340 measured reflections
5279 independent reflections
3971 reflections with *I* > 2σ(*I*)
*R*_int_ = 0.020

#### Refinement

*R*[*F* ^2^ > 2σ(*F* ^2^)] = 0.052
*wR*(*F* ^2^) = 0.166
*S* = 1.03
5279 reflections
250 parameters
80 restraints
H-atom parameters constrained
Δρ_max_ = 0.93 e Å^−3^
Δρ_min_ = −0.48 e Å^−3^

### 

Data collection: *SMART* (Bruker, 2000 ▶); cell refinement: *SAINT* (Bruker, 2000 ▶); data reduction: *SAINT*; program(s) used to solve structure: *SHELXS97* (Sheldrick, 2008 ▶); program(s) used to refine structure: *SHELXL97* (Sheldrick, 2008 ▶); molecular graphics: *X-SEED* (Barbour, 2001 ▶); software used to prepare material for publication: *publCIF* (Westrip, 2010 ▶).

## Supplementary Material

Crystal structure: contains datablocks global, I. DOI: 10.1107/S1600536810002825/hb5312sup1.cif

Structure factors: contains datablocks I. DOI: 10.1107/S1600536810002825/hb5312Isup2.hkl

Additional supplementary materials:  crystallographic information; 3D view; checkCIF report

## Figures and Tables

**Table:** 

Zn1—S4	2.3256 (11)
Zn1—S1	2.3375 (11)
Zn1—S2	2.3434 (10)
Zn1—S3	2.3560 (10)

**Table:** 

S4—Zn1—S1	130.64 (5)
S4—Zn1—S2	129.21 (5)
S1—Zn1—S2	77.59 (4)
S4—Zn1—S3	77.52 (3)
S1—Zn1—S3	126.46 (5)
S2—Zn1—S3	123.05 (5)

**Fig. 1.**
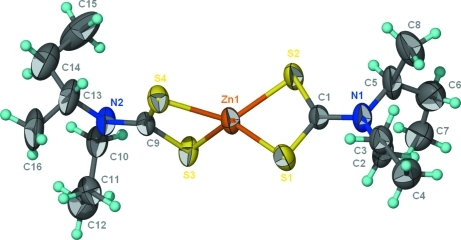
View of (I) at the 50% probability level; hydrogen atoms are drawn as spheres of arbitrary radius. The disorder is not shown.

## supplementary crystallographic information

### Experimental

Zinc chloride (10 mmol), *i*-butyl-*n*-propylamine (20 mmol), carbon disulfide (20 mmol) and ammonia (10 ml) were reacted in ethanol (30 ml) at 277 K to produce a white solid. This was collected and recrystallized from ethanol to yield colourless blocks of (I).

### Refinement

The carbon atoms of the alkyl chains (except for the four atoms connected to the nitrogen atoms) show large displacement ellipsoids. The disorder could not be refined, and was assumed to be a 1:1 disorder. The 1,2-related carbon-carbon distances were restrained to 1.50±0.01 Å and the 1,3-related ones to 2.35±0.01 Å. The temperature factors of the primed atoms were set to those of the unprimed ones; the anisotropic temperature factors of all disordered atoms were restrained to be nearly isotropic.

Carbon-bound H-atoms were placed in calculated positions (C—H 0.93 to 0.98 Å) and were included in the refinement in the riding model approximation, with *U*(H) set to 1.2 to 1.5*U*(C).

### Crystal data

**Table:**

[Zn(C_8_H_16_NS_2_)_2_]	*F*(000) = 944
*M**_r_* = 446.05	*D*_x_ = 1.289 Mg m^−^^3^
Monoclinic, *P*2_1_/*n*	Mo *K*α radiation, λ = 0.71073 Å
Hall symbol: -P 2yn	Cell parameters from 4652 reflections
*a* = 14.2151 (7) Å	θ = 2.3–26.4°
*b* = 11.8527 (6) Å	µ = 1.43 mm^−^^1^
*c* = 15.1428 (7) Å	*T* = 293 K
β = 115.691 (1)°	Block, colourless
*V* = 2299.15 (19) Å^3^	0.30 × 0.30 × 0.25 mm
*Z* = 4	

### Data collection

**Table:**

Bruker SMART APEX diffractometer	5279 independent reflections
Radiation source: fine-focus sealed tube	3971 reflections with *I* > 2σ(*I*)
graphite	*R*_int_ = 0.020
ω scans	θ_max_ = 27.5°, θ_min_ = 1.6°
Absorption correction: multi-scan (*SADABS*; Sheldrick, 1996)	*h* = −18→9
*T*_min_ = 0.673, *T*_max_ = 0.716	*k* = −15→15
15340 measured reflections	*l* = −18→19

### Refinement

**Table:**

Refinement on *F*^2^	Primary atom site location: structure-invariant direct methods
Least-squares matrix: full	Secondary atom site location: difference Fourier map
*R*[*F*^2^ > 2σ(*F*^2^)] = 0.052	Hydrogen site location: inferred from neighbouring sites
*wR*(*F*^2^) = 0.166	H-atom parameters constrained
*S* = 1.03	*w* = 1/[σ^2^(*F*_o_^2^) + (0.0943*P*)^2^ + 1.1606*P*] where *P* = (*F*_o_^2^ + 2*F*_c_^2^)/3
5279 reflections	(Δ/σ)_max_ = 0.001
250 parameters	Δρ_max_ = 0.93 e Å^−^^3^
80 restraints	Δρ_min_ = −0.48 e Å^−^^3^

### Fractional atomic coordinates and isotropic or equivalent isotropic displacement parameters (Å^2^)

**Table:**

	*x*	*y*	*z*	*U*_iso_*/*U*_eq_	Occ. (<1)
Zn1	0.55521 (3)	0.44936 (4)	0.32988 (4)	0.06385 (19)	
S1	0.62441 (7)	0.27447 (8)	0.31726 (9)	0.0732 (3)	
S2	0.73544 (7)	0.48158 (9)	0.41144 (10)	0.0779 (3)	
S3	0.44152 (8)	0.56222 (9)	0.19904 (8)	0.0685 (3)	
S4	0.42531 (7)	0.49493 (10)	0.37820 (7)	0.0669 (3)	
N1	0.8309 (2)	0.2955 (3)	0.3903 (3)	0.0709 (9)	
N2	0.2993 (2)	0.6465 (3)	0.2508 (2)	0.0671 (8)	
C1	0.7415 (3)	0.3442 (3)	0.3754 (3)	0.0580 (8)	
C2	0.8334 (3)	0.1754 (3)	0.3595 (4)	0.0752 (11)	
H2A	0.7717	0.1602	0.2991	0.090*	0.50
H2B	0.8942	0.1640	0.3471	0.090*	0.50
H2C	0.7797	0.1663	0.2930	0.090*	0.50
H2D	0.9002	0.1621	0.3583	0.090*	0.50
C5	0.9327 (3)	0.3572 (4)	0.4397 (4)	0.0952 (17)	
H5A	0.9147	0.4355	0.4464	0.114*	0.50
H5B	0.9190	0.4349	0.4530	0.114*	0.50
C9	0.3786 (2)	0.5769 (3)	0.2728 (2)	0.0530 (7)	
C10	0.2641 (3)	0.7191 (4)	0.1610 (3)	0.0760 (12)	
H10A	0.3258	0.7484	0.1569	0.091*	0.50
H10B	0.2266	0.7831	0.1700	0.091*	0.50
H10C	0.3239	0.7399	0.1495	0.091*	0.50
H10D	0.2328	0.7877	0.1710	0.091*	0.50
C13	0.2460 (4)	0.6573 (5)	0.3161 (4)	0.1049 (19)	
H13A	0.2854	0.6105	0.3738	0.126*	0.50
H13B	0.2705	0.5973	0.3655	0.126*	0.50
C3	0.837 (2)	0.095 (2)	0.4378 (17)	0.077 (3)	0.50
H3A	0.7726	0.0996	0.4450	0.092*	0.50
H3B	0.8945	0.1146	0.5000	0.092*	0.50
C4	0.852 (5)	−0.0219 (13)	0.408 (4)	0.096 (4)	0.50
H4A	0.8554	−0.0746	0.4576	0.144*	0.50
H4B	0.9151	−0.0252	0.4002	0.144*	0.50
H4C	0.7937	−0.0409	0.3470	0.144*	0.50
C6	1.0024 (12)	0.362 (3)	0.3931 (11)	0.123 (3)	0.50
H6A	1.0437	0.2939	0.4067	0.148*	0.50
H6B	1.0496	0.4258	0.4186	0.148*	0.50
C7	0.9398 (14)	0.3752 (14)	0.2859 (9)	0.105 (3)	0.50
H7A	0.9844	0.3669	0.2535	0.157*	0.50
H7B	0.9082	0.4486	0.2722	0.157*	0.50
H7C	0.8864	0.3184	0.2627	0.157*	0.50
C8	1.002 (2)	0.315 (4)	0.5456 (14)	0.119 (4)	0.50
H8A	0.9752	0.3446	0.5894	0.179*	0.50
H8B	1.0722	0.3403	0.5658	0.179*	0.50
H8C	1.0004	0.2340	0.5469	0.179*	0.50
C11	0.196 (2)	0.6651 (19)	0.0648 (10)	0.072 (5)	0.50
H11A	0.1316	0.6390	0.0650	0.086*	0.50
H11B	0.2317	0.6010	0.0532	0.086*	0.50
C12	0.174 (4)	0.752 (3)	−0.0135 (9)	0.107 (4)	0.50
H12A	0.1281	0.7209	−0.0759	0.160*	0.50
H12B	0.2380	0.7750	−0.0148	0.160*	0.50
H12C	0.1412	0.8165	0.0001	0.160*	0.50
C14	0.2359 (12)	0.7698 (14)	0.353 (2)	0.129 (4)	0.50
H14A	0.2099	0.7630	0.4026	0.155*	0.50
H14B	0.1882	0.8167	0.3003	0.155*	0.50
C15	0.3433 (17)	0.8197 (16)	0.3967 (19)	0.159 (14)	0.50
H15A	0.3411	0.8939	0.4211	0.239*	0.50
H15B	0.3684	0.8243	0.3473	0.239*	0.50
H15C	0.3893	0.7729	0.4495	0.239*	0.50
C16	0.1365 (9)	0.6086 (15)	0.268 (2)	0.137 (5)	0.50
H16A	0.1388	0.5297	0.2829	0.206*	0.50
H16B	0.1086	0.6182	0.1980	0.206*	0.50
H16C	0.0927	0.6471	0.2917	0.206*	0.50
C3'	0.818 (2)	0.087 (2)	0.4221 (18)	0.077 (3)	0.50
H3'A	0.7452	0.0858	0.4115	0.092*	0.50
H3'B	0.8609	0.1027	0.4908	0.092*	0.50
C4'	0.847 (5)	−0.0254 (13)	0.395 (4)	0.096 (4)	0.50
H4'A	0.8375	−0.0832	0.4353	0.144*	0.50
H4'B	0.9183	−0.0237	0.4060	0.144*	0.50
H4'C	0.8028	−0.0410	0.3275	0.144*	0.50
C6'	0.9843 (14)	0.359 (3)	0.3750 (12)	0.123 (3)	0.50
H6'A	0.9950	0.2825	0.3586	0.148*	0.50
H6'B	1.0519	0.3951	0.4078	0.148*	0.50
C7'	0.9189 (15)	0.4218 (13)	0.2844 (9)	0.105 (3)	0.50
H7'A	0.9572	0.4327	0.2462	0.157*	0.50
H7'B	0.9005	0.4939	0.3014	0.157*	0.50
H7'C	0.8565	0.3796	0.2470	0.157*	0.50
C8'	1.010 (2)	0.303 (4)	0.5354 (15)	0.119 (4)	0.50
H8'A	1.0595	0.2579	0.5228	0.179*	0.50
H8'B	0.9735	0.2557	0.5614	0.179*	0.50
H8'C	1.0471	0.3607	0.5820	0.179*	0.50
C11'	0.185 (3)	0.656 (3)	0.0723 (14)	0.099 (9)	0.50
H11C	0.2139	0.5847	0.0640	0.119*	0.50
H11D	0.1221	0.6407	0.0803	0.119*	0.50
C12'	0.161 (4)	0.731 (3)	−0.0147 (9)	0.107 (4)	0.50
H12D	0.1163	0.6919	−0.0734	0.160*	0.50
H12E	0.2249	0.7511	−0.0180	0.160*	0.50
H12F	0.1268	0.7981	−0.0082	0.160*	0.50
C14'	0.2732 (13)	0.7661 (19)	0.365 (2)	0.129 (4)	0.50
H14C	0.2412	0.8241	0.3156	0.155*	0.50
H14D	0.2416	0.7708	0.4101	0.155*	0.50
C15'	0.373 (2)	0.791 (2)	0.414 (2)	0.199 (15)	0.50
H15D	0.3804	0.8643	0.4425	0.298*	0.50
H15E	0.4056	0.7901	0.3698	0.298*	0.50
H15F	0.4063	0.7359	0.4645	0.298*	0.50
C16'	0.1275 (11)	0.6532 (18)	0.263 (2)	0.137 (5)	0.50
H16D	0.1013	0.7264	0.2363	0.206*	0.50
H16E	0.0996	0.6312	0.3075	0.206*	0.50
H16F	0.1069	0.5993	0.2102	0.206*	0.50

### Atomic displacement parameters (Å^2^)

**Table:**

	*U*^11^	*U*^22^	*U*^33^	*U*^12^	*U*^13^	*U*^23^
Zn1	0.0423 (2)	0.0630 (3)	0.0817 (3)	0.01003 (17)	0.0227 (2)	0.0059 (2)
S1	0.0483 (5)	0.0569 (5)	0.1016 (8)	0.0001 (4)	0.0205 (5)	−0.0099 (5)
S2	0.0443 (5)	0.0598 (5)	0.1181 (9)	0.0049 (4)	0.0245 (5)	−0.0227 (6)
S3	0.0601 (5)	0.0842 (7)	0.0756 (6)	0.0210 (5)	0.0428 (5)	0.0234 (5)
S4	0.0554 (5)	0.0868 (7)	0.0610 (5)	0.0156 (5)	0.0274 (4)	0.0203 (5)
N1	0.0508 (16)	0.0522 (16)	0.112 (3)	0.0086 (13)	0.0378 (18)	−0.0017 (17)
N2	0.0561 (16)	0.085 (2)	0.0686 (18)	0.0238 (16)	0.0346 (15)	0.0199 (16)
C1	0.0472 (17)	0.0521 (18)	0.074 (2)	0.0042 (14)	0.0253 (16)	−0.0016 (16)
C2	0.070 (2)	0.061 (2)	0.110 (3)	0.0088 (19)	0.053 (2)	−0.006 (2)
C5	0.049 (2)	0.066 (3)	0.169 (5)	0.0063 (18)	0.045 (3)	−0.014 (3)
C9	0.0409 (15)	0.0626 (19)	0.0573 (18)	0.0045 (14)	0.0231 (14)	0.0057 (15)
C10	0.067 (2)	0.076 (3)	0.091 (3)	0.026 (2)	0.040 (2)	0.024 (2)
C13	0.093 (3)	0.144 (5)	0.102 (4)	0.056 (4)	0.065 (3)	0.027 (3)
C3	0.066 (8)	0.062 (4)	0.100 (6)	0.009 (4)	0.033 (6)	−0.002 (4)
C4	0.102 (6)	0.066 (3)	0.121 (9)	0.008 (3)	0.049 (7)	−0.002 (4)
C6	0.096 (6)	0.138 (5)	0.146 (7)	−0.031 (5)	0.063 (6)	−0.049 (6)
C7	0.100 (6)	0.123 (9)	0.104 (4)	0.000 (6)	0.056 (4)	−0.014 (5)
C8	0.070 (4)	0.091 (8)	0.160 (6)	0.011 (4)	0.015 (4)	0.005 (6)
C11	0.059 (7)	0.073 (8)	0.081 (11)	0.004 (6)	0.028 (8)	0.018 (7)
C12	0.113 (8)	0.110 (10)	0.092 (3)	0.031 (7)	0.040 (3)	0.027 (4)
C14	0.138 (9)	0.146 (7)	0.131 (6)	0.049 (7)	0.084 (8)	0.011 (5)
C15	0.25 (3)	0.081 (9)	0.24 (3)	−0.047 (15)	0.19 (3)	−0.053 (13)
C16	0.083 (4)	0.202 (12)	0.158 (7)	0.048 (6)	0.082 (5)	0.022 (9)
C3'	0.066 (8)	0.062 (4)	0.100 (6)	0.009 (4)	0.033 (6)	−0.002 (4)
C4'	0.102 (6)	0.066 (3)	0.121 (9)	0.008 (3)	0.049 (7)	−0.002 (4)
C6'	0.096 (6)	0.138 (5)	0.146 (7)	−0.031 (5)	0.063 (6)	−0.049 (6)
C7'	0.100 (6)	0.123 (9)	0.104 (4)	0.000 (6)	0.056 (4)	−0.014 (5)
C8'	0.070 (4)	0.091 (8)	0.160 (6)	0.011 (4)	0.015 (4)	0.005 (6)
C11'	0.081 (15)	0.137 (19)	0.072 (10)	0.023 (11)	0.025 (9)	0.027 (10)
C12'	0.113 (8)	0.110 (10)	0.092 (3)	0.031 (7)	0.040 (3)	0.027 (4)
C14'	0.138 (9)	0.146 (7)	0.131 (6)	0.049 (7)	0.084 (8)	0.011 (5)
C15'	0.19 (2)	0.19 (3)	0.17 (2)	0.03 (2)	0.041 (17)	−0.085 (19)
C16'	0.083 (4)	0.202 (12)	0.158 (7)	0.048 (6)	0.082 (5)	0.022 (9)

### Geometric parameters (Å, °)

**Table:**

Zn1—S4	2.3256 (11)	C7—H7C	0.9600
Zn1—S1	2.3375 (11)	C8—H8A	0.9600
Zn1—S2	2.3434 (10)	C8—H8B	0.9600
Zn1—S3	2.3560 (10)	C8—H8C	0.9600
S1—C1	1.719 (4)	C11—C12	1.498 (8)
S2—C1	1.732 (4)	C11—H11A	0.9700
S3—C9	1.717 (3)	C11—H11B	0.9700
S4—C9	1.736 (3)	C12—H12A	0.9600
N1—C1	1.323 (4)	C12—H12B	0.9600
N1—C5	1.499 (5)	C12—H12C	0.9600
N1—C2	1.504 (5)	C14—C15	1.498 (7)
N2—C9	1.318 (4)	C14—H14A	0.9700
N2—C13	1.490 (5)	C14—H14B	0.9700
N2—C10	1.499 (5)	C15—H15A	0.9600
C2—C3'	1.496 (9)	C15—H15B	0.9600
C2—C3	1.503 (9)	C15—H15C	0.9600
C2—H2A	0.9700	C16—H16A	0.9600
C2—H2B	0.9700	C16—H16B	0.9600
C2—H2C	0.9700	C16—H16C	0.9600
C2—H2D	0.9700	C3'—C4'	1.499 (10)
C5—C6	1.447 (9)	C3'—H3'A	0.9700
C5—C6'	1.457 (9)	C3'—H3'B	0.9700
C5—C8'	1.533 (9)	C4'—H4'A	0.9600
C5—C8	1.556 (9)	C4'—H4'B	0.9600
C5—H5A	0.9800	C4'—H4'C	0.9600
C5—H5B	0.9800	C6'—C7'	1.480 (10)
C10—C11	1.498 (8)	C6'—H6'A	0.9700
C10—C11'	1.524 (19)	C6'—H6'B	0.9700
C10—H10A	0.9700	C7'—H7'A	0.9600
C10—H10B	0.9700	C7'—H7'B	0.9600
C10—H10C	0.9700	C7'—H7'C	0.9600
C10—H10D	0.9700	C8'—H8'A	0.9600
C13—C14'	1.452 (17)	C8'—H8'B	0.9600
C13—C14	1.477 (7)	C8'—H8'C	0.9600
C13—C16	1.518 (7)	C11'—C12'	1.50 (2)
C13—C16'	1.521 (16)	C11'—H11C	0.9700
C13—H13A	0.9800	C11'—H11D	0.9700
C13—H13B	0.9800	C12'—H12D	0.9600
C3—C4	1.502 (9)	C12'—H12E	0.9600
C3—H3A	0.9700	C12'—H12F	0.9600
C3—H3B	0.9700	C14'—C15'	1.32 (2)
C4—H4A	0.9600	C14'—H14C	0.9700
C4—H4B	0.9600	C14'—H14D	0.9700
C4—H4C	0.9600	C15'—H15D	0.9600
C6—C7	1.482 (10)	C15'—H15E	0.9600
C6—H6A	0.9700	C15'—H15F	0.9600
C6—H6B	0.9700	C16'—H16D	0.9600
C7—H7A	0.9600	C16'—H16E	0.9600
C7—H7B	0.9600	C16'—H16F	0.9600
			
S4—Zn1—S1	130.64 (5)	H6A—C6—H6B	108.3
S4—Zn1—S2	129.21 (5)	C6—C7—H7A	109.5
S1—Zn1—S2	77.59 (4)	C6—C7—H7B	109.5
S4—Zn1—S3	77.52 (3)	H7A—C7—H7B	109.5
S1—Zn1—S3	126.46 (5)	C6—C7—H7C	109.5
S2—Zn1—S3	123.05 (5)	H7A—C7—H7C	109.5
C1—S1—Zn1	83.12 (12)	H7B—C7—H7C	109.5
C1—S2—Zn1	82.68 (12)	C5—C8—H8A	109.5
C9—S3—Zn1	82.61 (12)	C5—C8—H8B	109.5
C9—S4—Zn1	83.14 (11)	H8A—C8—H8B	109.5
C1—N1—C5	121.4 (3)	C5—C8—H8C	109.5
C1—N1—C2	120.6 (3)	H8A—C8—H8C	109.5
C5—N1—C2	118.0 (3)	H8B—C8—H8C	109.5
C9—N2—C13	120.7 (3)	C10—C11—C12	107.6 (8)
C9—N2—C10	120.1 (3)	C10—C11—H11A	110.2
C13—N2—C10	119.2 (3)	C12—C11—H11A	110.2
N1—C1—S1	121.5 (3)	C10—C11—H11B	110.2
N1—C1—S2	122.1 (3)	C12—C11—H11B	110.2
S1—C1—S2	116.37 (19)	H11A—C11—H11B	108.5
C3'—C2—N1	116.0 (13)	C11—C12—H12A	109.5
C3—C2—N1	110.6 (13)	C11—C12—H12B	109.5
C3—C2—H2A	109.5	H12A—C12—H12B	109.5
N1—C2—H2A	109.5	C11—C12—H12C	109.5
C3'—C2—H2B	114.5	H12A—C12—H12C	109.5
C3—C2—H2B	109.5	H12B—C12—H12C	109.5
N1—C2—H2B	109.5	C13—C14—C15	105.9 (8)
H2A—C2—H2B	108.1	C13—C14—H14A	110.5
C3'—C2—H2C	108.3	C15—C14—H14A	110.5
C3—C2—H2C	119.5	C13—C14—H14B	110.5
N1—C2—H2C	108.3	C15—C14—H14B	110.5
C3'—C2—H2D	108.3	H14A—C14—H14B	108.7
C3—C2—H2D	102.2	C14—C15—H15A	109.5
N1—C2—H2D	108.3	C14—C15—H15B	109.5
H2A—C2—H2D	116.5	H15A—C15—H15B	109.5
H2C—C2—H2D	107.4	C14—C15—H15C	109.5
C6—C5—N1	119.6 (13)	H15A—C15—H15C	109.5
C6'—C5—N1	109.3 (13)	H15B—C15—H15C	109.5
C6'—C5—C8'	106.0 (8)	C13—C16—H16A	109.5
N1—C5—C8'	114.3 (18)	C13—C16—H16B	109.5
C6—C5—C8	104.9 (8)	H16A—C16—H16B	109.5
C6'—C5—C8	114.9 (12)	C13—C16—H16C	109.5
N1—C5—C8	113.5 (17)	H16A—C16—H16C	109.5
C6—C5—H5A	105.9	H16B—C16—H16C	109.5
C6'—C5—H5A	106.5	C2—C3'—C4'	108.4 (9)
N1—C5—H5A	105.9	C2—C3'—H3'A	110.0
C8'—C5—H5A	114.5	C4'—C3'—H3'A	110.0
C8—C5—H5A	105.9	C2—C3'—H3'B	110.0
C6—C5—H5B	107.4	C4'—C3'—H3'B	110.0
C6'—C5—H5B	109.1	H3'A—C3'—H3'B	108.4
N1—C5—H5B	109.1	C3'—C4'—H4'A	109.5
C8'—C5—H5B	109.1	C3'—C4'—H4'B	109.5
C8—C5—H5B	100.5	H4'A—C4'—H4'B	109.5
N2—C9—S3	121.9 (3)	C3'—C4'—H4'C	109.5
N2—C9—S4	121.9 (3)	H4'A—C4'—H4'C	109.5
S3—C9—S4	116.21 (19)	H4'B—C4'—H4'C	109.5
N2—C10—C11	117.2 (9)	C5—C6'—C7'	109.5 (9)
N2—C10—C11'	110.2 (12)	C5—C6'—H6'A	109.8
N2—C10—H10A	108.0	C7'—C6'—H6'A	109.8
C11—C10—H10A	108.0	C5—C6'—H6'B	109.8
C11'—C10—H10A	117.5	C7'—C6'—H6'B	109.8
N2—C10—H10B	108.0	H6'A—C6'—H6'B	108.2
C11—C10—H10B	108.0	C6'—C7'—H7'A	109.5
C11'—C10—H10B	105.5	C6'—C7'—H7'B	109.5
H10A—C10—H10B	107.2	H7'A—C7'—H7'B	109.5
N2—C10—H10C	109.6	C6'—C7'—H7'C	109.5
C11—C10—H10C	100.3	H7'A—C7'—H7'C	109.5
C11'—C10—H10C	109.6	H7'B—C7'—H7'C	109.5
H10B—C10—H10C	113.8	C5—C8'—H8'A	109.5
N2—C10—H10D	109.6	C5—C8'—H8'B	109.5
C11—C10—H10D	111.3	H8'A—C8'—H8'B	109.5
C11'—C10—H10D	109.6	C5—C8'—H8'C	109.5
H10C—C10—H10D	108.1	H8'A—C8'—H8'C	109.5
C14'—C13—N2	108.3 (12)	H8'B—C8'—H8'C	109.5
C14—C13—N2	119.4 (12)	C12'—C11'—C10	106.4 (15)
C14'—C13—C16	125.1 (10)	C12'—C11'—H11C	110.5
C14—C13—C16	106.2 (7)	C10—C11'—H11C	110.5
N2—C13—C16	110.9 (13)	C12'—C11'—H11D	110.5
C14'—C13—C16'	106.8 (9)	C10—C11'—H11D	110.5
N2—C13—C16'	114.1 (13)	H11C—C11'—H11D	108.6
C14—C13—H13A	106.5	C11'—C12'—H12D	109.5
N2—C13—H13A	106.5	C11'—C12'—H12E	109.5
C16—C13—H13A	106.5	H12D—C12'—H12E	109.5
C16'—C13—H13A	122.0	C11'—C12'—H12F	109.5
C14'—C13—H13B	109.2	H12D—C12'—H12F	109.5
C14—C13—H13B	115.2	H12E—C12'—H12F	109.5
N2—C13—H13B	109.2	C15'—C14'—C13	117.8 (17)
C16'—C13—H13B	109.2	C15'—C14'—H14C	107.9
C2—C3—C4	108.0 (9)	C13—C14'—H14C	107.9
C2—C3—H3A	110.1	C15'—C14'—H14D	107.9
C4—C3—H3A	110.1	C13—C14'—H14D	107.9
C2—C3—H3B	110.1	H14C—C14'—H14D	107.2
C4—C3—H3B	110.1	C14'—C15'—H15D	109.5
H3A—C3—H3B	108.4	C14'—C15'—H15E	109.5
C3—C4—H4A	109.5	H15D—C15'—H15E	109.5
C3—C4—H4B	109.5	C14'—C15'—H15F	109.5
H4A—C4—H4B	109.5	H15D—C15'—H15F	109.5
C3—C4—H4C	109.5	H15E—C15'—H15F	109.5
H4A—C4—H4C	109.5	C13—C16'—H16D	109.5
H4B—C4—H4C	109.5	C13—C16'—H16E	109.5
C5—C6—C7	109.0 (9)	H16D—C16'—H16E	109.5
C5—C6—H6A	109.9	C13—C16'—H16F	109.5
C7—C6—H6A	109.9	H16D—C16'—H16F	109.5
C5—C6—H6B	109.9	H16E—C16'—H16F	109.5
C7—C6—H6B	109.9		
			
S4—Zn1—S1—C1	−134.68 (14)	Zn1—S4—C9—N2	−173.3 (3)
S2—Zn1—S1—C1	−3.07 (14)	Zn1—S4—C9—S3	6.65 (19)
S3—Zn1—S1—C1	119.05 (14)	C9—N2—C10—C11	80.2 (17)
S4—Zn1—S2—C1	135.98 (14)	C13—N2—C10—C11	−102.0 (17)
S1—Zn1—S2—C1	3.05 (14)	C9—N2—C10—C11'	87.6 (19)
S3—Zn1—S2—C1	−122.60 (14)	C13—N2—C10—C11'	−94.6 (19)
S4—Zn1—S3—C9	4.50 (13)	C9—N2—C13—C14'	108.3 (12)
S1—Zn1—S3—C9	136.26 (13)	C10—N2—C13—C14'	−69.4 (12)
S2—Zn1—S3—C9	−124.42 (13)	C9—N2—C13—C14	125.1 (12)
S1—Zn1—S4—C9	−132.20 (13)	C10—N2—C13—C14	−52.7 (12)
S2—Zn1—S4—C9	118.25 (13)	C9—N2—C13—C16	−111.0 (10)
S3—Zn1—S4—C9	−4.45 (13)	C10—N2—C13—C16	71.3 (11)
C5—N1—C1—S1	179.1 (3)	C9—N2—C13—C16'	−132.9 (10)
C2—N1—C1—S1	−2.0 (6)	C10—N2—C13—C16'	49.3 (11)
C5—N1—C1—S2	0.1 (6)	C3'—C2—C3—C4	65 (14)
C2—N1—C1—S2	179.0 (3)	N1—C2—C3—C4	−174 (3)
Zn1—S1—C1—N1	−174.6 (4)	C6'—C5—C6—C7	13 (12)
Zn1—S1—C1—S2	4.5 (2)	N1—C5—C6—C7	38 (3)
Zn1—S2—C1—N1	174.6 (4)	C8'—C5—C6—C7	161 (3)
Zn1—S2—C1—S1	−4.5 (2)	C8—C5—C6—C7	167 (3)
C1—N1—C2—C3'	−74.6 (14)	N2—C10—C11—C12	−178 (3)
C5—N1—C2—C3'	104.4 (14)	C11'—C10—C11—C12	136 (18)
C1—N1—C2—C3	−85.3 (14)	C14'—C13—C14—C15	6(6)
C5—N1—C2—C3	93.7 (14)	N2—C13—C14—C15	−51 (3)
C1—N1—C5—C6	−127.5 (15)	C16—C13—C14—C15	−177 (2)
C2—N1—C5—C6	53.5 (15)	C16'—C13—C14—C15	−168 (3)
C1—N1—C5—C6'	−122.5 (12)	C3—C2—C3'—C4'	−104 (14)
C2—N1—C5—C6'	58.5 (12)	N1—C2—C3'—C4'	−167 (3)
C1—N1—C5—C8'	118.9 (15)	C6—C5—C6'—C7'	−140 (17)
C2—N1—C5—C8'	−60.0 (15)	N1—C5—C6'—C7'	63 (3)
C1—N1—C5—C8	107.7 (16)	C8'—C5—C6'—C7'	−174 (3)
C2—N1—C5—C8	−71.2 (16)	C8—C5—C6'—C7'	−168 (3)
C13—N2—C9—S3	179.9 (4)	N2—C10—C11'—C12'	−176 (3)
C10—N2—C9—S3	−2.4 (5)	C11—C10—C11'—C12'	−38 (14)
C13—N2—C9—S4	−0.2 (6)	C14—C13—C14'—C15'	176 (11)
C10—N2—C9—S4	177.6 (3)	N2—C13—C14'—C15'	−54 (4)
Zn1—S3—C9—N2	173.4 (3)	C16—C13—C14'—C15'	172 (3)
Zn1—S3—C9—S4	−6.58 (18)	C16'—C13—C14'—C15'	−178 (3)
